# Parental migration and left-behind children in Georgia – school teachers’ experience and perception: a qualitative study

**DOI:** 10.1186/s12889-022-14516-8

**Published:** 2022-11-14

**Authors:** Khatia Antia, Astrid Berner Rodoreda, Volker Winkler

**Affiliations:** Heidelberg Institute of Global Health, University Hospital Heidelberg, Im Neuenheimer Feld 130.3, 69120 Heidelberg, Germany

**Keywords:** Left-behind Children, Migration, Labour, Parents, Schoolteachers, Education, Health, Well-being

## Abstract

**Introduction:**

Georgia, like other Eastern European countries, showed a dramatic increase of international labour emigration after becoming independent in 1991. The collapse of the Soviet Union caused economic instability, unemployment and poverty resulting in labour migration. Since then, thousands of children have been left behind in the care of extended family members while their parents work abroad. The aim of this study is to explore schoolteachers’ perceptions on parental migration and left-behind children (LBC) in Georgia as schoolteachers are the main contact persons for LBC and their caregivers.

**Methods:**

We conducted six focus-group discussions with public school teachers, namely class-tutors and six in-depth interviews with school principals from two migrant sending regions. We applied reflexive thematic analysis to systematically analyse the data and identify main and sub-themes. The contextual model of family stress underpins this study.

**Results:**

We identified the following themes expressed by both, teachers, and school principals: social and economic impact of migrant labour and relationships between schools and migrant families. School teachers and principals acknowledged some positive aspects of migrant labour, but primarily perceived parental migration as a negative experience for children leading to problems in mental health, well-being, and academic performance. Structural factors, lack of support and lack of community involvement were expressed to further worsen the situation. Teachers saw themselves as one of the main supporters for LBC while they described the role of caregivers ranging from caring to unhelpful or even destructive. School principals stated mitigating the situation by regular meetings with class tutors, extra-tutoring for LBC, psychological counselling, and developing/enacting internal guidelines.

**Conclusions:**

Our findings suggest that LBCs and transnational families could benefit from the provision of psychological services at schools.

## Introduction

Georgia is an Eastern European Post-Soviet country, which showed a dramatic increase of international emigration after becoming independent in 1991. Following the independence, unemployment, poverty and economic instability pushed people to seek working opportunities abroad [1, 2]. According to The State Commission on Migration Issues [3] in 2002, its population had decreased by 20% compared to 1989; from 5.4 million people when Georgia was still a member of the Soviet Union to 4.3 million people barely more than a decade later. Between 2002 and 2015, the population further decreased by 600,000 people, largely due to temporary labour migration [3].

Following to the population decrease, remittances sent by labour migrants increased by 64% in 2020, showing highest increase in the last two decades [1]. Mostly remittances are sent from Russia, Italy, Greece, The US, Israel and Turkey [4]. Despite a sharp decline in Russian residence permits for Georgians (52% from 2017 to 2020) the biggest share of remittances still comes from Russia [1, 4]. With respect to the EU, Italy, Greece, Germany and Spain are leading countries from which Georgians send remittances [1, 4]. A cost and benefit analysis of labour mobility between the EU and the Eastern Partnership countries estimated that by 2050 Georgia will be the country with the fourth largest population loss [5]. Due to economic instability, most temporary migration exceeds the planned period of 3–6 months and follows a continuous cycle of working abroad and returning home.

Labour migrants usually migrate alone, while their family members (including children and spouses) remain in countries of origin. In literature, families divided across borders are called *transnational families*, while the notion itself is called *transnationalism* [6–8]*. Left-behind children* (LBC) is the term commonly used to address the left children of both, internal and international migrant workers [9–11]. Another term increasingly used in transnational family literature is *staying behind children* [12–15].

Transnational family literature emphasizes profound impact, especially psychological and stress related outcomes of transnationalism on household members [6–8, 16–21]. To investigate stress in the context of left-behind children, the following theoretical frameworks are used: attachment theory, theory of ambiguous loss and the contextual model of family stress [19, 21]. According to the contextual model of family stress, stressful life events, daily hassles and other stressors may put families into crises in which the family as a system loses its balance [22, 23]. Parental labour migration is considered as a potential stressor associated with adverse mental health outcomes for transnational families and especially for the left-behind children [9, 24].

In Georgia, more than 57% of migrants are female and almost 39% of all children are affected by migratory separation, which is the highest percentage in the Eastern-European region [4, 5]. Furthermore, recent studies suggest that children affected by migratory separation are at increased risk of developing physical and mental health disorders [10, 24–32]. The number of Georgian children with mental and behavioural disorders increased four-times between 2013 and 2019 (from 1.1 to 4.4 per 100,000 children) [33]. Even though the Georgian Ministry of Health and other Public Health Institutions state the promotion of children’s health and well-being as one of their main priorities, the needs of LBC seem not to be addressed sufficiently.

Existing literature emphasizes the importance of social structure, school community and teachers’ engagement in supporting children [34–39]. School teachers, and especially class tutors are seen to play an enabling role as they are frequently in contact with LBC and their caregivers and endeavour to improve the situation according to the children’s needs. According to the order №06 /n of the Ministry of Education of Georgia, each class has one of the schoolteachers assigned as class tutor [40]: in addition to teaching, the class tutor has the legal authority and obligation to ensure safety, to regularly monitor children’s school life and to communicate with families [40]. Yet, schools in Georgia hardly receive guidance on how to effectively decrease the vulnerability of LBC.

Internationally, several studies investigated the effects of parental migration on children from the perspective of children, parents, and caregivers and mostly observed worsened mental health and well-being outcomes, lowered academic performance, increased vulnerability, inadequate care, and lack of support [17, 18, 25, 39, 41, 42]. In this study, we explore schoolteachers’ perspective and perceptions about left-behind children (LBC) and their families in a quest to better adapt supportive interventions. To our knowledge, this is the first study targeting schoolteachers in the context of LBC research in Georgia. Understanding the needs of LBC and their families is essential for developing supportive targeted interventions.

## Methods

### Study setting and participants

We conducted this qualitative exploratory study in the regions Samegrelo-Zemo Svaneti and Guria in the western part of Georgia. Both regions were selected because of their high out-migration rates [4, 43–45]. In each region, we selected three public schools from urban and rural areas. To include both rural and urban communities, we selected schools showing a range in the number of students (38 to 478) with estimated percentages of LBC from 15 to 50%. We conducted six individual in-depth interviews (IDI) with the school principals and six focus group discussions (FGD) with 7–12 teachers. The reason to focus on school principals and class tutors was their responsibility in assisting students with academic and social problems, mediating between students, parents and teachers, organizing regular meetings, and informing parents about their child's behavior and academic performance [40, 46].

### Sampling and data collection

Data collection was performed during October–November 2019 by the data collection team namely the interviewer (K.A.) and two research assistants (N.A., S.G.). All principals of the selected schools agreed to participate in the study. For FGDs, we performed purposive sampling to select participants by principal’s invitation to all class tutors. During the initial meeting, we informed potential participants about the purpose and procedures of the study, distributed study information and consent forms and clarified questions asked. We arranged FGDs after one week of the meeting. Participation was voluntarily and written consent was collected. Considering the availability and due to the relatively small number of class tutors in each school (up to 20 in bigger schools from urban areas) we accepted all participants willing to take part in the FGD. Each FGD lasted approximately 50–80 min and was performed in classrooms at the respective schools after the regular working hours. IDIs with school principals were conducted in their offices and lasted about 35–45 min.

FGDs and IDIs were semi-structured and conducted in Georgian, which is the native language of the interviewer, the research assistants, and all participants. All data collection was audio-recorded, transcribed, and translated to English by the data collection team. Additionally, the team took reflexive notes and conducted debriefing after each interview/discussion as suggested by McMahon and Winch [47] to enhance the quality of the collected data.

Focus group discussions covered the following topics: children’s behaviour, school performance, emotional stability, self-esteem, caregiver’s involvement in school activities, children’s relationship with their caregivers, teachers’ attitude towards LBC; while in-depth interviews covered the following ones: perceived impact of parental migration on LBC, measures from school administration to support LBC, actions or initiatives to address the needs of children affected by migratory separation. In line with Morse [48, 49], interviewing stopped when no new relevant information was obtained, and saturation was reached.

### Analysis

The contextual model of family stress underpins this study.

We applied thematic analysis [50, 51] to identify main themes and sub-themes from the collected data and used a combination of inductive and deductive coding. Deductive coding was based on the interview guide. Thematic analysis within a constructivist framework enabled us to explain, analyse, synthesise deeper levels of identified topics, concepts and ideas as social constructs [52, 53].

We performed the analysis in the following four phases: first, familiarisation with transcribed data. Second, after carefully reading the interviews multiple times, the first and the second authors identified themes. The first author performed systematic coding by using QSR International’s NVivo version 12. This included coding the same interviews several times in a systematic manner to bring out various dimensions such as social and economic impact of migrant labour, complexity of relationship between schools and migrant parents/caregivers. Reflexive thematic analysis is built on the idea of deep inductive exploration [50, 54] and does not include multiple data coders. The complex nature of the topic motivated us to involve all authors in discussing the developing codebook and the themes initially identified. Based on the familiarity with the transcripts, all authors were involved in further theme development which was based on several cycles of regrouping codes and guided by Braun, Clarke [50] principles of reflexive thematic analysis. After reviewing, refining, and analysing each theme, at the final phase, we contextualized the analytic narrative in relation to available literature.

The study was approved by the ethics committee of the Medical Faculty Heidelberg N-S-652/2019 and the Ministry of Education in Georgia. For ease of presentation, all participants will be referenced by an identification pseudonym composed of (i) type of research (FGD or IDI), (ii) school ID (A to F), and (iii) for teachers, individual IDs (running number). This study followed the main principles of Consolidated Criteria for Reporting Qualitative research (COREQ) [55].

## Results

Table 1 shows detailed characteristics of the selected schools. Since the school administration has no requirement to monitor and document the number of LBCs, the percentages were estimated by the respective school principal.

**Table 1:** School characteristics including estimated percentage of left-behind children

School ID	A	B	C	D	E	F
School size [N students]	478	92	118	627	114	38
LBC estimated %	30%	20%	15%	20%	15%	50%
Area	urban	rural	urban	urban	rural	rural
Region	SZS^a^	SZS	G^b^	G	SZS	G

*SZS*^a^- Samegrelo-Zemo Svaneti region, *G*^b^ Guria region

We conducted six FGDs, among which three consisted of women only, while another three were mixed. In each FGD, the number of participants varied between 7 and 12, their age ranged from 30 to 69 years (mean age 51). All, except one interviewed school principal were women, with the age ranging from 56 to 74 years. Furthermore, school principals were on average 11 years older than the FGD participants. Years of practice as a school principal ranged from 4 to 27. Most participants were women reflecting the gender distribution of teachers and principals in Georgia.

With respect to the major hosting countries of parents, the schoolteachers, as well as principals named Italy, Turkey, Greece, Poland, Israel, Russia and Ukraine. Due to the absence of official statistics on LBC and labour migrants per school/region, these results reflect the views of the participants and may not show the exact picture of migration trajectories of transnational families in Georgia.

After examining the data, we conceptualized a thematic map with two main and several subthemes (see Table 2): 1. Social and economic impact of migrant labour; 2. Relationships between schools and migrant families. In the following, we provide a narrative overview of each theme and subtheme.

**Table 2:** Main themes with the respective subthemes derived from thematic analysis

Main Theme	Sub-theme
1. Social and economic impact of migrant labour	1. Physical and mental health impact of separation
	2. Impact of gender on separation and academic performance
	3. Role of schools – care-substitutes versus caregivers
	4. Economic impact – materialism
	5. Long separation of parents leading to divorce
2. Relationships between Schools and Migrant families	1. Disrupted communication and high expectations by parents towards teachers
	2. Schools in need of external support

## Theme 1: Social and economic impact of migrant labour

This section presents key aspects of migratory separation on children as seen by the class tutors and school principals. It gives an overview of the following dimensions of social and economic impact of migrant labour: physical and psychological health; academia; age and gender of a child at separation; caregivers’ and schools’ roles in relation to the well-being of LBC; material benefits, and other social impact.

### Physical and mental health impact of separation

In class tutors’ and principals’ views, parental absence negatively affects children’s psychological health and nutrition. The participants argued that LBC may gradually develop mental health issues such as melancholy and sadness, anxiety, aggression, and depression.

FGD participants shared similar observations on how LBC’s lives change after migratory separation. The following quote by IDI_D pictures such change:

“*He was such a talented, well-disciplined child, but separation overshadowed everything. He became aggressive. Even involvement of the police was necessary and currently he is under the constant supervision of the school and the police”.*

In the tutors’ views, younger children seem to be more open to communicate their feelings and needs; the older they get, the more distant they become. The class tutors found it challenging to communicate with adolescents. One of the participants described her impression as follows:

*“When they are little, they miss their parents a lot. They need warmth. They come and hug us all the time, they seek mothers in us, but when they get older, they try so hard not to show it, they lock-themselves inside.”* (FGD_ A_ 9)

Through the analysis, we identified migrant parents’ gender as a factor affecting LBC’s physiological health. Most interviewed school principals and class tutors described children of migrant mothers as more vulnerable than children of migrant fathers. In their view, children see mothers as role-models; after migratory separation, mothers sometimes become an inspiration for children, they even paint mother’s portraits in art classes. One of the FGD participants stressed this aspect:

*“When the mother is helping a child with class materials, the child sees the God in her. In the child’s perception, the mother can do anything, “she can help me solve math problems, and she can help me do my homework” – thinks a child and now, in such a situation, most of our pupils suffer from the separation with the mother.”* (FGD_D_3)

However, class tutors as well as principals agreed in their perception that, irrespective of the gender of the migrating parent, LBC perceive migratory separation as painful. In principals’ views, children having both parents migrating suffer the most. As described by female participant of IDI_B: *“When both parents leave, the child literally remains an orphan.”* Despite modern digital communication technologies enabling children to talk to their parent every day, the participants consider parents’ physical presence as crucial for the well-being of their children.

With respect to physical health, most of the FGD participants identified poor nutrition due to unhealthy eating habits as a risk among LBC. According to class tutors, some LBC tend to use the remittances for buying and bringing unhealthy food (sugary drinks and snacks) to school. This concern was more evident among participants from urban schools. None of the participants irrespective of location mentioned poor nutrition in the context of not having enough food or children being malnourished. Rather, the opposite was the case: participants seemed concerned about LBC’s food choices leading to unhealthy nutrition instead of investing in nutritious food. The teachers consider lack of control on the money LBC bring to school as dangerous since they may destroy their own health and set a bad example for their classmates.

### Impact of gender on separation and academic performance

Daughters of migrating mothers were said to need the help of their grandmothers for cooking, cleaning, and caregiving responsibilities for younger siblings. Sons of migrant fathers were seen as taking on typical male tasks such as farming and harvesting, which results in frequently missing school. Lacking supervision of fathers was another concern; especially adolescents were said to get involved in risky, unhealthy behaviour, such as smoking, alcohol consumption and gambling.

All interviewed school principals and most of the FGD participants suggested that academic performance suffers among LBC. The participants considered parental absence to be the cause of lower school grades. However, some participants argued that children from well-disciplined families, and children having supportive, relatively young caregivers, still manage to study well. All participants highlighted that on parent(s) return, LBC’s academic performance rapidly improves.

### Role of schools’ care-substitutes versus caregivers

We conceptualized school principals and schoolteachers (including class tutors) as mediators and supporters for LBC and their transnational families. Interviewed school principals reported the following strategies to support: regular teacher meetings dedicated to LBC’s issues; identifying children in need of additional academic support; communicating with caregivers and parents; involving the police and the social service agency when problems escalate and cannot be solved at the school level. To highlight this, one of the participants shared:

“*We, teachers feel what the problems are and try to resolve them that something fatal does not happen. We use different techniques. We also try to read psychological literature and provide psychological help to these children. Today, this is the foundation of our success. All of us do this individually, and by sharing with each other”.* (FGD_A_9*)*

FGD as well as IDI participants considered their actions and supportive activities as effective. They assumed that LBC feel better in the school environment than in other settings.

Class tutors described caregivers (mostly grandparents) as supportive. However, they considered caregivers’ age as an important contributor to the quality of provided care. Due to chronic diseases, increased workload, and other hardships, older grandparents were believed to not get fully involved in child’s upbringing. This may in teachers’ opinion lead to LBC’s increased stress, worsened health, and lower academic performance. The tutors recognized among LBC the fear that elderly grandparents may die due to stress. FGD_ B_3 recalled her communication with LBC:

*““Teacher, you know, I could not sleep all night, because my grandmother was sick, and I was afraid” told me a girl of a migrant mother. […] the child thinks of her future; she does not know who will take care of her if the grandmother dies”.*

Overall, participants saw children’s relationships with their caregivers as friendly and warm. However, they also reported experiencing conflicts and communication difficulties, such as, adolescents’ refusal to be supervised by their grandparents. Grandparents were believed to find it difficult to deal with adolescents and were said to often call schools for support.

One of the school principals also recalled violence case when a caregiver (uncle) punished a boy who wanted to get married at the age of 17. This violence led to adolescent fleeing from home and the police had to be called in to solve the problem.

### Economic impact – materialism

In this section, we focus on economic aspects of parental migration and its influence on LBC as seen by class tutors and principals.

The class tutors from centralized, urban schools reported that children of migrants are materially better-off than their classmates which may to some extent compensate the loss caused by separation. One of the tutors’ commented:

*“They suffer a lot, but their basic needs are met; they have better living conditions now than they had before. The material benefits gradually bring ‘sweetness’ that they don't feel that much pain when they grow up.”* (FGD_ C_7)

By contrast, FGD participants from the rural schools do not see noticeable economic benefits of parents’ migration on their LBC. Most migrant parents were said to perform low-skilled and low-paid jobs, however, class tutors described families living in the villages as extremely poor. Remittances were thought to mainly pay-back multiple bank-loans.

In the FGD, participants expressed the view that LBC, who see material benefits from parents’ migration tend to get accustomed to receiving regular money. FGD_ A_4 said: “*Although children are unhappy, they are suffering for mother's absence, after some time, the mother becomes a money-making machine for them.”*

Child labour was another issue. School principals and tutors emphasized that some LBC prioritize work and wealth over education. Class tutors stated that migrant parents often take their adolescent children with them as short-term migrant workers; sometimes not even informing schools about the absence. Some school principals were concerned about adolescents losing out on education, and not being mature enough to perform such work.

Our analysis showed that labour migration may be seen as a ‘desirable future prospect’ by LBC. Class tutors considered this as a danger. They argued that even left-behind adolescents, may lose motivation to study and follow their parents’ footsteps and become labour migrants themselves. Among LBC, the principals saw a lack of awareness towards the difficulties of leaving family members behind and performing heavy physical work.

### Long separation of parents leading to divorce – socio-economic impact

All participants perceived labour migration in Georgia as a strategy to survive extreme poverty; however, they feared it may lead to social disaster. A participant described this aspect as follows:

“*This is a social disaster that we are having in our country. Maybe in other countries, too, but mostly in our country. This is the destroying force for our society. We don’t want to make drama out of it, but this is a real tragedy.”* (FGD_ A_2)

In FGDs, participants expressed the view that poverty is not the only reason for parental migration. They highlighted that some mothers may migrate to avoid conflict with their spouses while long separation may lead to divorce. In the opinion of most tutors’ children of divorced migrant households suffer the most.

## Theme 2: Relationships between schools and migrant families

This section introduces the second theme we identified through data analysis of IDIs and FGDs: relationship challenges between schools and transnational families and schools’ perceived support of LBC’s needs. Additionally, the principals provided details on measures by the school administration to support LBC, and on actions or initiatives to address the needs of children affected by migratory separation.

### Disrupted communication and high expectations by parents towards teachers

Our findings showed that transnationalism usually comes with communication challenges between schools and migrant parents. Principals’ and tutors said they are hardly able to talk to the migrant parents even though they put a lot of effort and try different channels**.** They depicted many parents undervalue their role and importance as a mother or father and expect the school to take full responsibility for their children. Hence, the school principals perceived disrupted communication as an obstacle to implement supportive activities for LBC. One of the school principals described this issue as follows:

*“Children obviously have certain mental health issues, poor socio-economic conditions added to that. They need help; however, it is often difficult to communicate with parents, we try to connect them via Facebook, send messages almost every day, however, some never respond.”* (IDI _D)

### Schools in need of external support

All interviewed school principals stated that registration and monitoring of child being left-behind by governmental authorities as an urgent need:

*“We have had such discussions at the local resource centre. Especially among high school students, we have many cases of missing at school, and these are mostly children whose mothers or fathers are migrants. We have talked about this issue, but the representatives of the Ministry or any other organization have not addressed this issue and have not expressed interest.”* (IDI _B)

Several school principals raised the fact that the Georgian language does not have a specific term for ‘left-behind children’ and suggested generating one for registration purposes which also would help avoiding referring to LBC ‘such children’ during discussions.

None of the participating schools had a doctor, a nurse, or a psychologist among their staff. The Social Service Agency was the only organization, teachers across participating schools found helpful. All school principals emphasized the need of psychological counselling for LBC and teachers in schools.

Teachers called for actions in supporting LBC and their transnational families. Despite the economic crisis, class tutors, as well as principals thought that in some cases labour migration of parents could be avoided. In the participants’ view, some parents do not recognize the potential negative influence of parental migration on children. It was mentioned that parents motivated by material well-being only, lose connection, closeness, and warmth with their children.

One of the principals emphasised the need for action:

“*Whether it is a state or a non-governmental organization, it does not matter, but we need an organization that will work with parents, on their education and awareness, their attitudes towards school and towards their children. A teacher gives instructions to children on what to do and how to do, but when we observe, we see it is not working; the family environment should also change.”* (IDI_E)

Overall, the study found that opinions of tutors and school principals agreed in most aspects. Both considered parental labour migration as a major problem in Georgia that needs urgent attention. They endorsed ideas of helping and empowering LBC and their families. Most tutors appreciated the efforts and initiatives principals take to support LBC.

Female and male participants also agreed on the most aspects. Female participants tended to perceive mothers’ migration generally more traumatizing for LBC, while male participants emphasized the importance of fathers’ presence for boys. One of the male tutors stressed that left-behind girls may develop aggression towards remaining fathers and blame them for their mother’s absence.

## Discussion

Georgian schoolteachers saw left-behind children and transnational families as a major and increasing problem. Despite well-needed material benefits, they agreed that parental migration is a negative experience in the life of most children. Even though school administrations and teachers claimed strong efforts and the use various strategies to support left-behind children, they reported neither being able to meet the children’s health nor educational needs. They considered additional external support to be crucial.

In this study, we considered parental labour migration as a potential stressor for transnational families and especially, for LBC. We conceptualised it using the model of family stress [23]. Our findings clearly showed the following aspects of the family stress model: (i) Parental migration as a stressful event(s); (ii) family’s resources in response to the stress – such as remaining parent’s and caregiver’s role in the well-being of LBC; (iii) the outcome – family’s perception (definition) of the event – family’s awareness on the effects of migratory separation on children, their material motivations, high expectations by parents towards teachers to fulfil role of parent; (iv) family crises: degree of a stress – the participants perceive parents’ migration as life event that may put families into crises even leading to conflicts and divorce.

Our study highlighted the complex nature of transnationalism and its impact on left-behind children. In the literature, growing-up with absent parents is referred to as *transnational childhood* [56]. Congruent with our findings, several systematic literature reviews suggest the following health issues to be prevalent among LBC: sadness, worries, anxiety, depression, loneliness, poor nutrition [9, 10, 24, 57, 58]. Separation and stress are also reflected in studies on LBC from Kyrgyzstan [59], from Sri Lanka [60], and from the Philippines [61]. The authors highlight altered family dynamics and emotional challenges due to parental migration. It should be noted that most of these studies are quantitative and based on LBC’s and caregivers’ self-reports. With respect to the geographical location, studies from China are prominent, where migration is mostly internal (from rural to urban areas) [62]. Unlike China, labour migration in Eastern European countries like Georgia is mainly international.

Studies from other Eastern European countries, such as Romania and Lithuania suggest only negative mental health outcomes among LBC [28, 63, 64]. In Georgia, however, studies also describe positive and non-differing psychological health outcomes between LBC and non-LBC [12, 65, 66]. For example Gassmann, Siegel [66] describe a higher well-being index among LBC in Georgia, while it did not differ in Moldova. These findings are partly in opposition to ours; schoolteachers see parental labour migration mainly as a negative life event and perceive LBC as particularly vulnerable to various health issues.

Several qualitative studies from various countries studied the perspective of LBC [16, 59, 67–71] and echo our findings: parental migration was seen as a traumatizing life experience, in China [71] even causing psychopathologies in Greece [70]. LBC in China [67], Ecuador [16] and Thailand [61] found good care and supportive caregivers to be important for a sense of stability, well-being and resilience; while inadequate care and structural factors such as divorce and poverty increased LBC’s vulnerability in China [71].

Our study participants considered LBC prone to poor nutrition and unhealthy eating habits. Scholars from China, explored this issue from the perspective of parents and caregivers (grandparents) and found intergenerational differences in beliefs about healthy eating [69]. As most of the LBC in Georgia are under the care of their grandparents, like China, the eating habits might also be influenced by caregivers believes.

The economic impact of parental migration was another aspect our study highlighted. The findings suggest that migrant families from urban areas are materially better off than those living in rural areas, while rural LBC and Non-LBC are perceived as equally poor. Unlike our findings, LBC believe they are materially better-off than peers from non-migrant households in the Philippines [72]. Economic conditions in host countries and the type of work parents perform may play a major role in this. Not all migrants send the same remittances and not all transnational families have the same financial needs. These differences underline the complexity and importance of context in terms of culture, tradition, family norms, and care-giving practice among transnational families across countries and continents.

With respect to the education, our findings were heterogeneous. While some participants expressed concerns about a worsening academic performance of LBC, others argued that children from supportive, well-disciplined families perform well. Our study identified economic crisis as the main motivation for labour migration, while scholars from China and the Philippines mention children’s education as the main motivation [73, 74]. However, at the time the authors describe negative effects of migrant labour on children’s education [73].

Our findings call for attention to the challenges schoolteachers are facing as care-substitutes. Critelli, Lewis [59] explain this with traditional cultural expectations of transnational families in post-Soviet Kyrgyzstan. Some parents’ attitude further worsens the situation and may lead to disrupted communication and conflicts, consequently leaving LBC’s health and other needs unmet.

Our study findings highlight the need for external organizations to work with, and to ensure better, clearer communication between LB children, their families, and schools. In line with our findings, Zhang [68] calls policymakers in China for the protection of LBC’s rights, such as right to provision, protection and participation. In another study Siriwardhana, Wickramage [60] underlines the need of health and social protection policy for Sri Lanka and for other migrant sending countries.

Despite teachers’ perceived challenges and vulnerabilities, our findings also show some positive aspects of migrant labour. Teachers from urban schools notice the economic benefits, such as improved living conditions, gradually helping children to cope better with parental absence. Participants see migration as a strategy to survive extreme poverty in rural Georgia. Overall, a good relationship and closeness with caregivers and with remaining family members could also be seen as advantages. In terms of education, not all LBC seem to be affected negatively; in teachers’ views, some LBC study well. Making parents happy with their academic achievements could be a motivation for some LBC. These aspects of migrant labour should be acknowledged.

Finally, our findings emphasize the positive influence schools have in the well-being of LBC. Yet, addressing the issue of LBC seems to be viewed as nonessential by other organizations operating around children’s issues in Georgia. State and non-governmental agencies should re-evaluate these assumptions because of the high importance and potential benefits of supportive actions for schools, for LBC and for their families.

## Limitation

Our results reflect the views of public-school teachers and principals from two out-migration regions and are therefore not necessarily representative for other regions. All findings are based on tutors’ and principals’ perceptions of left-behind children, their parents, and caregivers and therefore cannot be interpreted objectively. To get a comprehensive picture, data from children, their parents, and caregivers are needed. This would enable exploring important aspects such as gender (LB girl and LB boys, maternal, paternal and both parent migration), parents’ migration profile (host countries, length of migration, remittances), communication with migrant parents, self-reported well-being, and educational outcomes of children. We acknowledge that teachers’ and children’s perceptions on parental migration might differ. Considering that children are the main actors in transnational families, further studies exploring children’s own experiences and perspectives are needed. Despite these limitations, our qualitative study provides in-depth analysis of schoolteachers’ perceptions and thus adds a different perspective and more nuanced findings on how LBCs are perceived. We will extend this study to children and their perspectives will be provided in future articles.

## Conclusions

Schoolteachers perceive labour migration as a survival strategy for transnational families to overcome extreme poverty. Overall, left-behind children are described to have good a relationship with their caregivers. Yet, schoolteachers consider most left-behind children as particularly vulnerable to mental health problems. Factors, like communication difficulties between schools and migrant families is thought to further exacerbate children’s vulnerability. Schoolteachers feel the pressure to act as care-substitutes for LBC. However, they seem overwhelmed by this task and urgently call for support and action on the worsening situation of LBC.

Based on schoolteachers’ claim to be a supportive institution for LBC and their families, our study concludes that future partnerships to develop school/community-based interventions may benefit LBC’s health and well-being. A supportive environment and system may empower not only LBC, but also their migrant parents and transnational families.

Based on our findings we recommend that schools receive intensive training courses by child psychologists and social workers on early detection of LBC’s needs and effective assistance. LBC, as well as teachers, may benefit from targeted interventions such as psychological counselling services at schools.

Finally, our findings highlight the need of raising public awareness on the potential health and other risks of migratory separation on LBC. Actions to provide working opportunities within country seems essential to decrease child-parent separations and to mitigate negative consequences of parental absence on LBC. We further encourage public health scientists to put more emphasis on this topic and to explore various aspects of this complex issue.

## Data Availability

Collected data is stored in password protected hard drive at Heidelberg University Hospital. To protect confidentiality of participants datasets generated and/or analysed during the current study are not publicly available but are available from the corresponding author on reasonable request.

## Abbreviations

LBC: Left-behind children
FGD: Focus group Discussion
IDI: Individual In-depth Interview
TA: Thematic Analysis
COREQ: Consolidated criteria for reporting qualitative research
